# Supplementary material for: A Lévy expansion strategy optimizes early dune building by beach grasses
Source: Nat Commun. 2019 Jun 14;10:2656. doi: 10.1038/s41467-019-10699-8 (PMC6572860; doi:10.1038/s41467-019-10699-8)
Supplement: Supplementary file 1 — Supplementary Information [file 41467_2019_10699_MOESM1_ESM.pdf]

Supplementary Information

A Lévy expansion strategy optimizes early dune building by beach grasses

Reijers et al.

Supplementary Figures

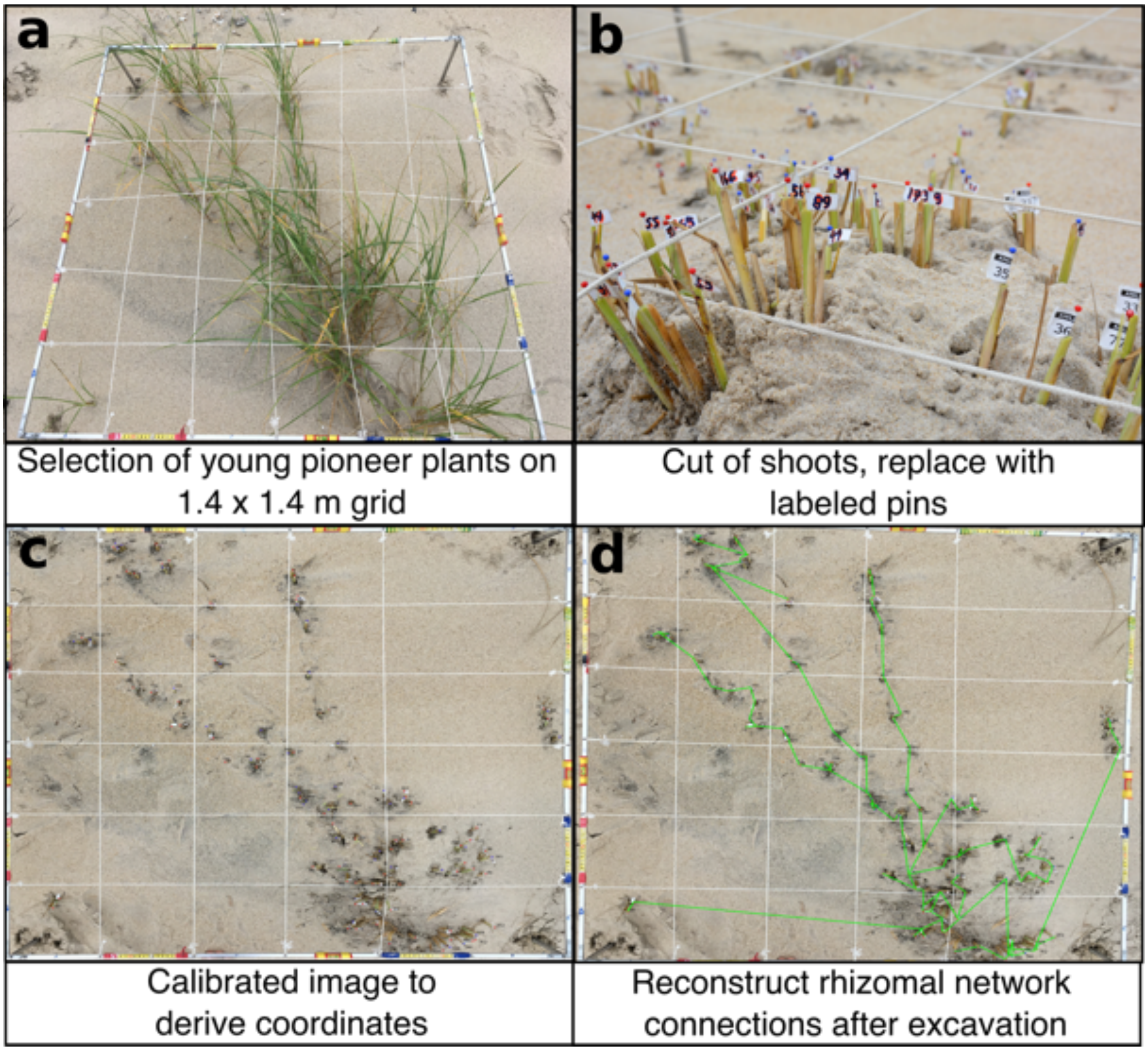

Supplementary Figure 1 | Picture overview showing the field method for deriving the step sizes between consecutive shoots in a clonal network. **a,** We selected young establishing plants on a 1.4 x 1.4 m grid growing on the front of the dune. **b,** All aboveground biomass was cut off and we placed a labelled, coloured pin at the shoot base. **c,** Using a calibrate image of the 1.4 x1.4 m grid, we were able to derive the spatial coordinates of all the shoots. **d,** The plants were excavated and their connections were written down to reconstruct their rhizomal network using Image J.

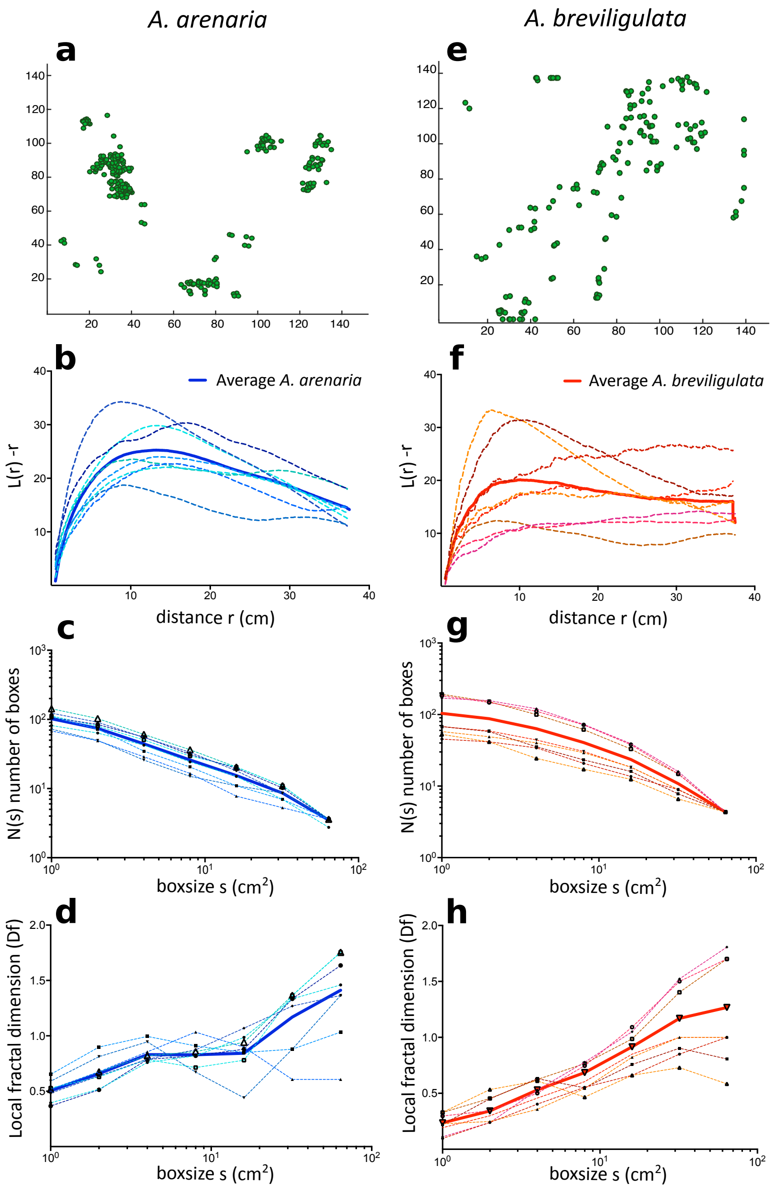

Supplementary Figure 2 | Analyses on the spatial organization of the shoots (N=8) of both *A. arenaria* and *A. breviligulata*. a,e, Depict the spatial organization of *A. arenaria* and *A. breviligulata* shoots, respectively. Point pattern analyses using the linearized Ripley's K: L(r), indicate strong clustering on the scale of 0-40 cm (L(r)>r) (thin lines represent separate plots and the thick line is the average per species) (**b,e**). Using boxcounting analyses we were able to determine whether the shoot organization of either species exhibited fractal properties (**c,g**). The fractal dimension (Df) of the shoot organization was Df~0.8 (flat line at ~2-16 cm) for *A. arenaria* (**d**) and *A. breviligulata* showed no signs of fractal properties (**h**). Source data are provided as a Source Data file.

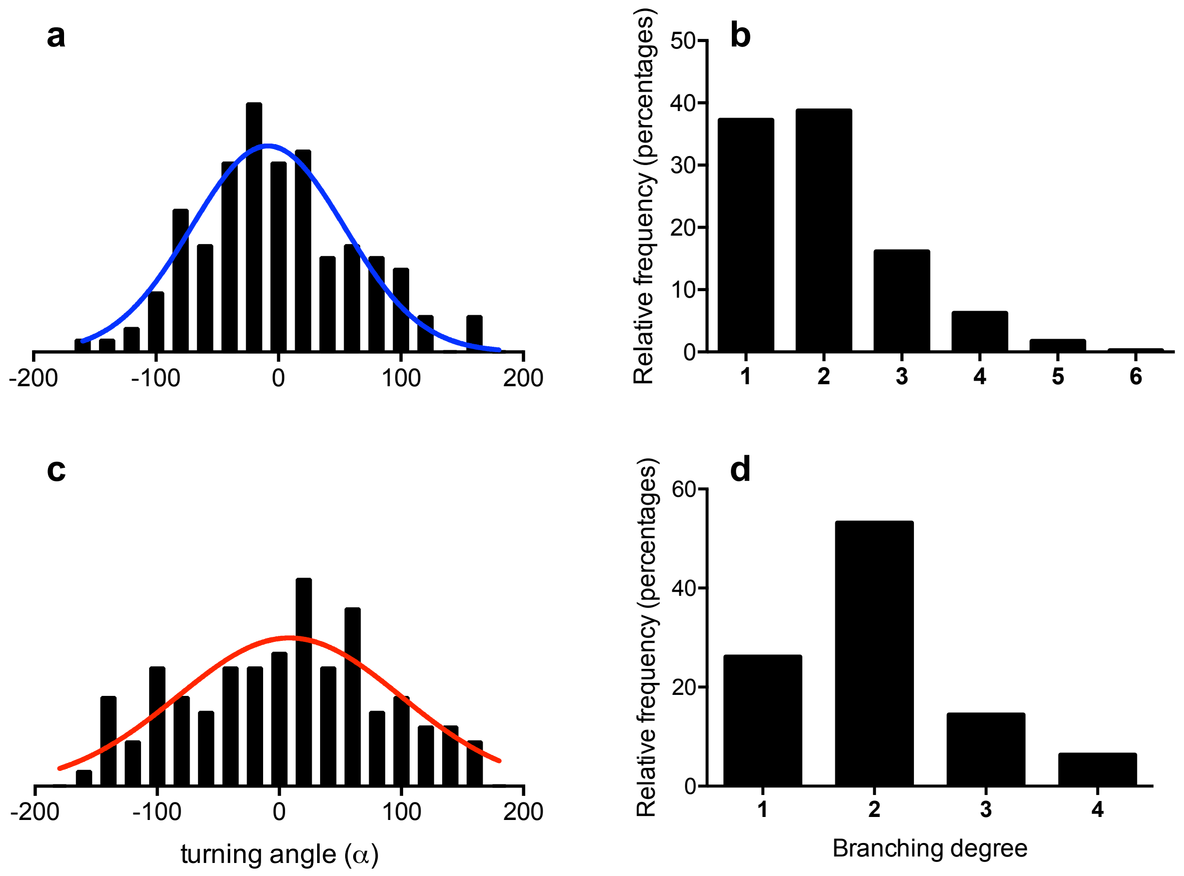

Supplementary Figure 3 | Frequency distribution of other field parameters. a,c Show the branching (turning) angles and **(b,d)** the branching degree. Figure depicts the distribution of *A. arenaria* (top) and *A. breviligulata* (bottom), respectively. Source data are provided as a Source Data file.

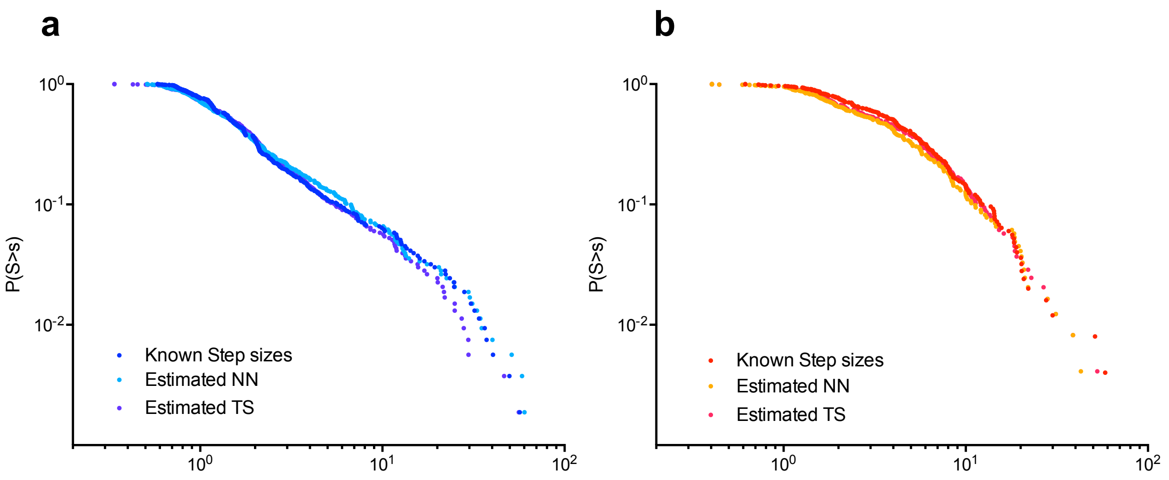

Supplementary Figure 4 | Inverse cumulative frequency distributions of the measured step sizes in the field and the estimated step sizes using the two connecting algorithms (nearest neighbour search (NN) and travelling salesman (TS)). a, Depicts the distribution of *A. arenaria* and **b**, the distribution of *A. breviligulata*. For both species and both connecting algorithms the null hypothesis based on a two-sample Kolmogorov-Smirnov test not rejected at the 5% significance level. For *A. arenaria:* NN vs. know step sizes (p=0.06), TS vs. known step sizes (p=0.11) and for *A. breviligulata*: NN vs. known step sizes (p=0.05), TS vs. known step sizes (p=0.13). Source data are provided as a Source Data file.

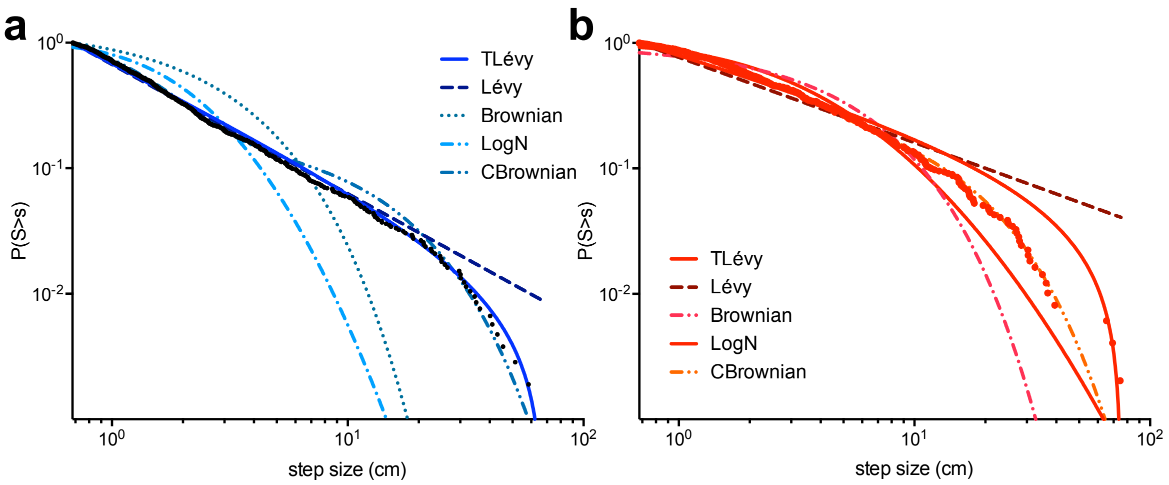

Supplementary Figure 5 | Inverse cumulative frequency distribution (e.g. the fraction of step sizes ≥ than a given step size s) and all fitted distribution functions for the total data set of both species. a, Depicts the pooled data set and the fitted distributions for *A. arenaria* (N= 1053) and **b,** depicts the pooled data and fitted distributions for *A. breviligulata* (N= 492). TLévy stands for truncated Pareto distribution, Lévy for an unbounded Pareto distribution, Brownian for an exponential distribution, LogN for a lognormal distribution and CBrownian for a two-mode exponential distribution. See methods for a description of fitting procedure. Source data are provided as a Source Data file.

a b

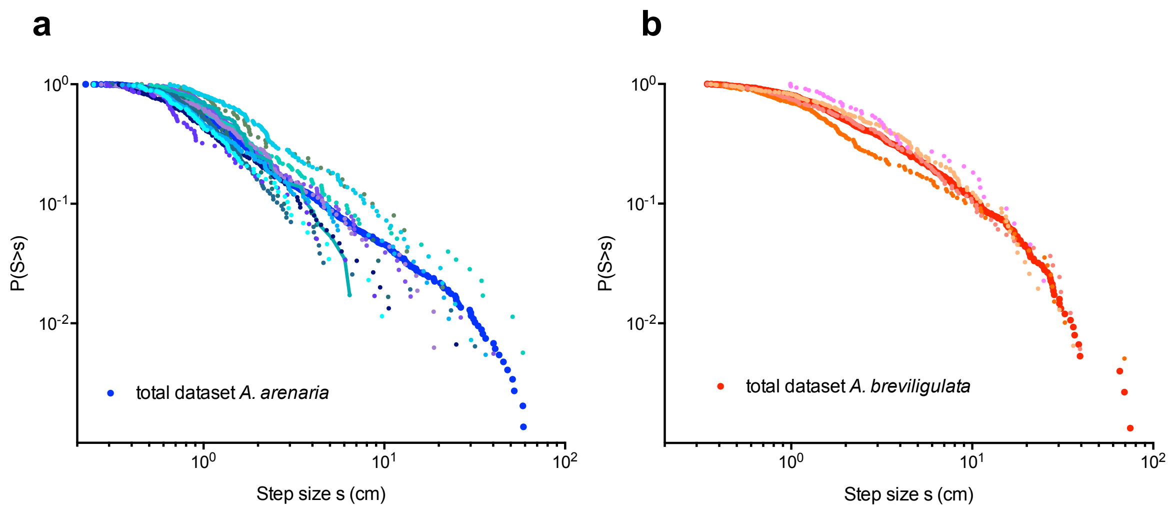

Supplementary Figure 6 | Inverse cumulative frequency distribution (e.g. the fraction of step sizes ≥ than a given step size s) for all the individual plants (N=8 for *A. arenaria* and N=4 for *A. breviligulata*). **a**, The thick blue markers indicate the distribution for the combined data of *A. arenaria* and for **b**, the tick red markers indicate the combined data for *A. breviligulata*. Source data are provided as a Source Data file.

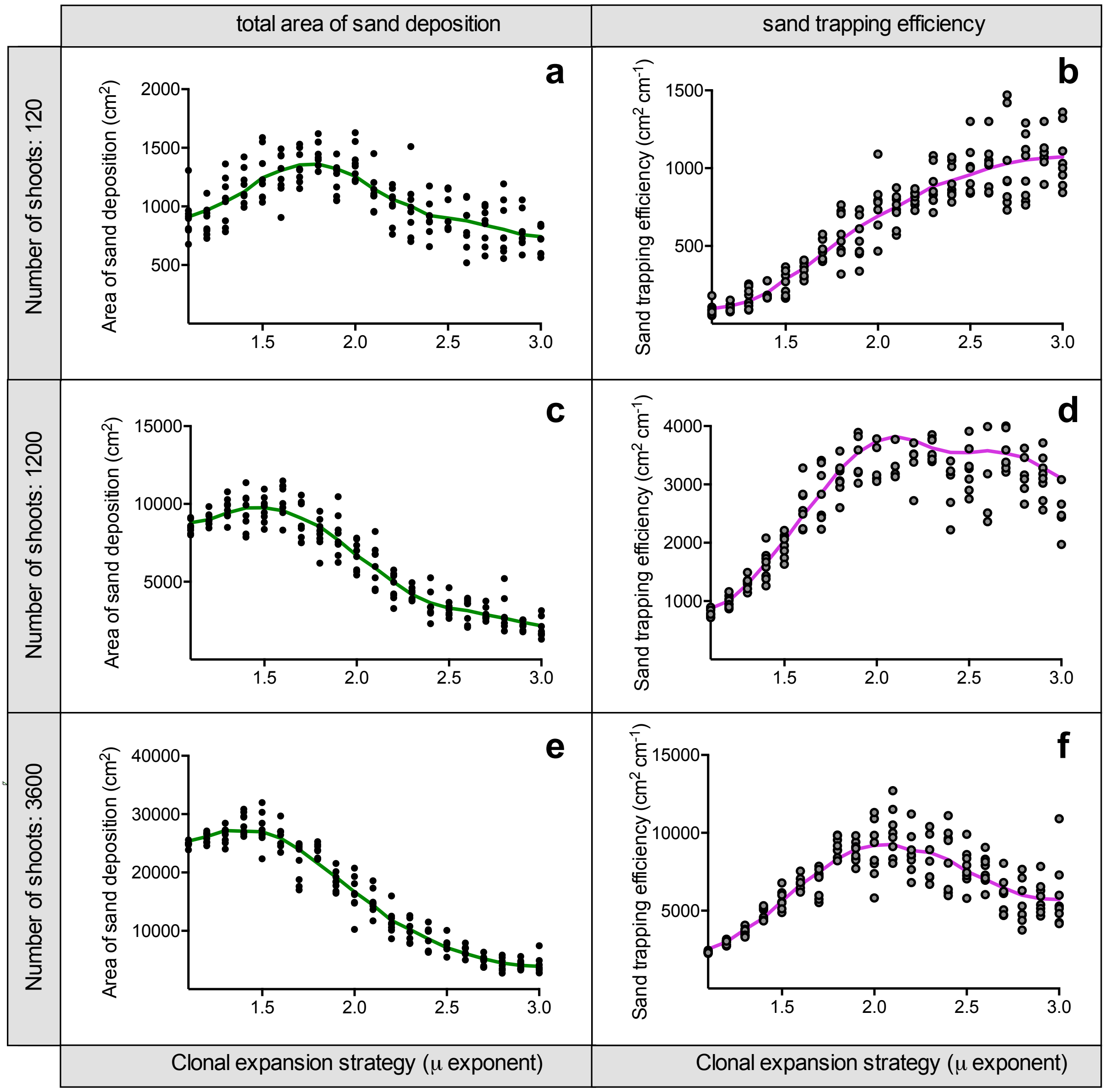

Supplementary Figure 7 | Model results (N=8) showing the relation between the clonal expansion strategy (μ exponent) and the biophysical feedback strength. a-e, Depict the total area of sand deposition and **b-f**, the sand trapping efficiency for three different shoot numbers (120, 1200 and 3600 shoots). Error bars represent ± SE. Source data are provided as a Source Data file.

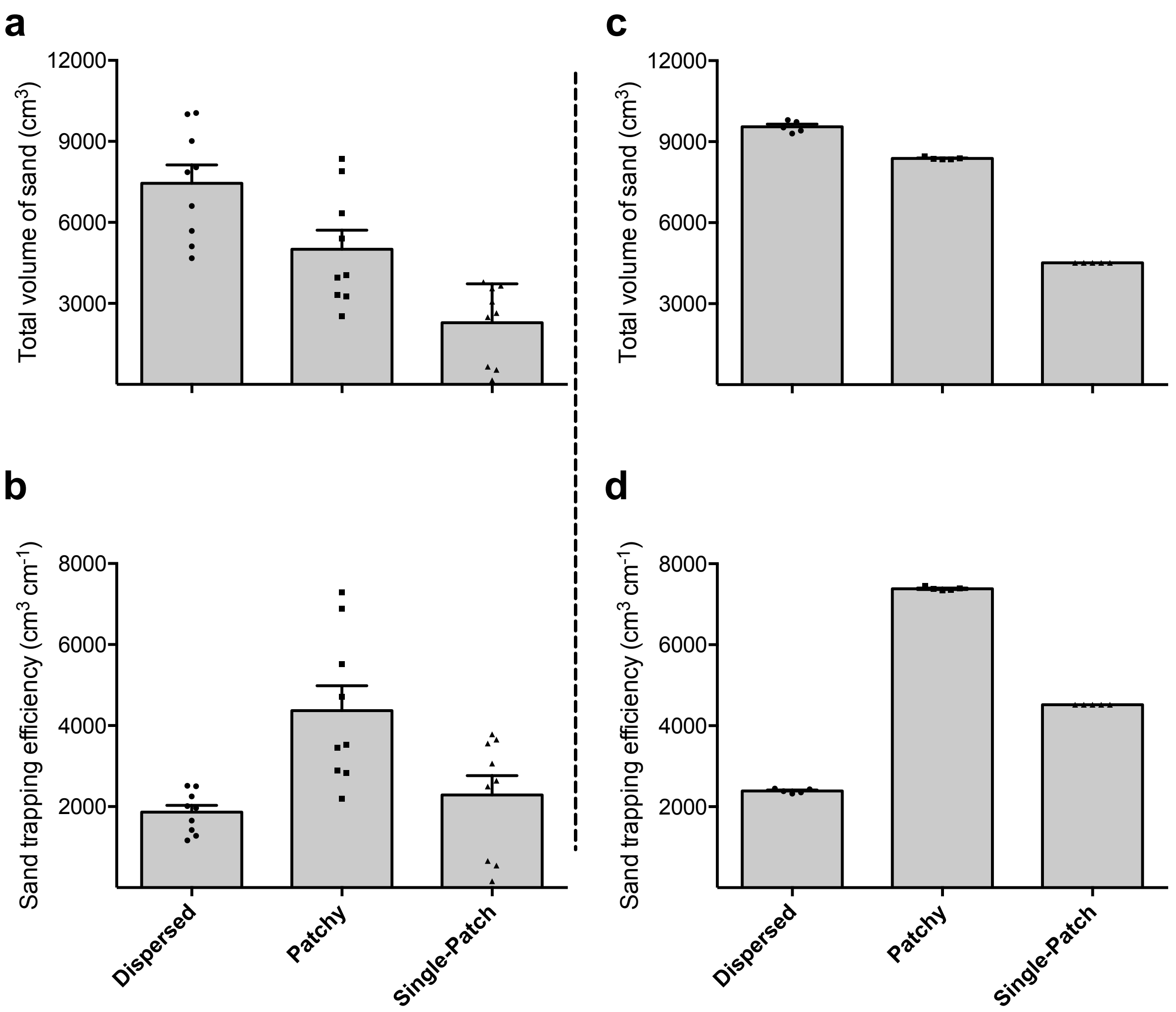

Supplementary Figure 8 | Comparison between experimental results (left side) and model simulations (right side). a & b, Depict the total volume of sand and the sand trapping efficiency, respectively, as indicated in Figure 4 (N=3, with 3 times repeated measures). **c & d**, Depict the potential area of sand deposition and sand trapping efficiency, respectively, for the configurations we used in our experiment simulated in our biophysical model (N=5). Error bars represent ± SE. Source data are provided as a Source Data file.

Supplementary Tables

Supplementary Table 1 | Outcome of the best-supported model to describe the step size data of the pooled dataset and the individual clonal plants. Data were obtained using the Nearest Neighbour (NN) connecting algorithm and only individual plants were analysed for which sufficient data (N>30) was available. Model selection and verification were done based on the weighted AIC value (wAIC) and the two-sample Kolmogrov-Smirnov (KS) test (bold numbers indicate a rejection at the 5% significance level). The parameters values or the shape exponents of the distributions: μ (Lévy), Tμ (truncated Lévy) and the λ values and weight for the composite Brownian were obtained using likelihood estimates (see Methods for detailed description). Note that for most individual plants the different candidate models can not be rejected at the 5% significance level. However, the obtained parameter values identify clear differences between the two species, with the distribution of *A. arenaria* having a steeper slope (i.e. more patchy spatial organization) than *A. breviligulata*.

**Plant ID**	**Number of steps/ % steps <smin**	**Chosen model**	**parameters**	**Total distance**	**Smin / Smax**	**wAIC Brown**	**wAIC Lévy**	**wAIC TLévy**	**wAIC CBrown**	**wAIC LogN**	KS Lévy	KS tLévy	KS CBrown	KS LogN
Arenaria 1	60/ 31%	Lévy	μ = 2.24	130.6	0.69 / 22.74	<0.01	**0.74**	0.23	0.03	<0.01	**0.560**	**0.611**	**0.690**	0.002
Arenaria 2	71 26%	Lévy	μ = 1.95	284.0	0.68 / 43.14	<0.01	**0.61**	0.38	0.01	<0.01	**0.617**	**0.605**	**0.544**	<0.001
Arenaria 3	34 42%	Lévy	μ = 2.15	105.0	0.68 / 41.06	<0.01	**0.80**	0.19	<0.01	<0.01	**0.615**	**0.624**	**0.413**	0.001
Arenaria 4	127 36%	Lévy	μ = 2.36	297.6	0.68 / 45.58	<0.01	**0.77**	0.11	0.12	<0.01	**0.519**	**0.532**	**0.694**	<0.001
Arenaria 5	87 35%	Lévy	μ = 2.13	229.3	0.68 / 30.09	<0.01	**0.70**	0.30	<0.01	<0.01	**0.671**	**0.705**	**0.696**	<0.001

Arenaria<br>6	51<br>12%	CBrown	$\lambda_1 = 0.84$<br>$\lambda_2 = 0.11$<br>$w_1 = 0.95$	115.9	0.68 /<br>18.96	0.14	0.21	0.18	**0.43**	<0.01	**0.254**	**0.378**	**0.600**	0.001
Arenaria<br>7	77<br>24%	TLévy	Tμ = 1.77	236.9	0.68 /<br>18.85	<0.01	0.12	**0.85**	0.03	<0.01	**0.421**	**0.591**	**0.569**	<0.001
Arenaria<br>8	48<br>20%	Lévy	Tμ = 2.19	118.1	0.68 /<br>25.8	<0.01	**0.64**	0.19	0.17	<0.01	**0.460**	**0.499**	**0.623**	0.003
Arenaria<br>9	136<br>1%	CBrown	$\lambda_1 = 0.89$<br>$\lambda_2 = 0.12$<br>$w_1 = 0.73$	501.1	0.68 /<br>31.13	<0.01	<0.01	**0.31**	0.69	<0.01	**0.084**	**0.272**	**0.559**	<0.001
Arenaria<br>10	152<br>14%	Lévy	μ = 1.96	642.0	0.68 /<br>68.03	<0.01	**0.59**	0.40	<0.01	<0.01	**0.443**	**0.504**	**0.473**	<0.001
Arenaria<br>11	48<br>4%	TLévy	μ = 1.66	188.3	0.68 /<br>33.03	<0.01	0.30	**0.55**	0.14	<0.01	**0.423**	**0.507**	**0.622**	<0.001
Arenaria<br>12	162<br>9%	Lévy	μ = 2.05	483.3	0.68 /<br>60.32	<0.01	**0.62**	0.25	0.13	<0.01	**0.142**	0.023	**0.091**	<0.001
Total<br>*Arenaria*	1053<br>19%	TLévy	Tμ = 1.98		0.68 /<br>68.03	<0.01	<0.01	**0.67**	0.33	<0.01	0.011	0.028	**0.410**	<0.001
Breviligulata<br>1	44<br>0%	TLévy	Tμ = 1.49	261.1	0.98 /<br>36.77	0.01	0.08	**0.82**	0.10	<0.01	0.029	0.187	0.464	<0.001
Breviligulata<br>2	137<br>11%	CBrown	$\lambda_1 = 0.42$<br>$\lambda_2 = 0.09$<br>$w_1 = 0.69$	781.4	0.68 /<br>75.33	<0.01	<0.01	<0.01	**0.99**	0.01	0.021	0.157	0.693	<0.001
Breviligulata<br>3	145<br>12%	CBrown	$\lambda_1 = 0.49$<br>$\lambda_2 = 0.07$<br>$w_1 = 0.77$	768.3	0.68/<br>65.28	<0.01	<0.01	0.38	**0.61**	<0.01	**0.079**	**0.368**	<0.001	<0.001
Breviligulata<br>4	166<br>16%	CBrown	$\lambda_1 = 0.92$<br>$\lambda_2 = 0.08$<br>$w_1 = 0.74$	817.9	0.68/<br>74.70	<0.01	0.03	0.38	**0.59**	<0.01	**0.115**	**0.239**	**0.549**	<0.001
Total<br>*Breviligulata*	492<br>12%	CBrown	$\lambda_1 = 0.66$<br>$\lambda_2 = 0.07$<br>$w_1 = 0.79$		0.68 /<br>75.33	<0.01	<0.01	<0.01	**1.00**	<0.01	<0.001	0.032	0.619	<0.001

Supplementary Table 2 | Biogeochemical analyses of soil and leaf samples. Soil (N=8) and leaf samples (N=5) were collected during the field survey. Soil samples were taken at ~ 5 cm depth in the middle of the clonal individual. Data are expressed as mean ± SE. From the table it is visible that especially N availability is very low (~0 %N) in these sandy beach systems, resulting in low foliar N:P ratio's (10.2 vs. 6.5 for *A. arenaria* and *A. breviligulata*, respectively), with N:P ratios <10 indicative of N deficiency[1,2].

**Soil type**	**% Organic Matter** (g g^{-1})	**Olsen-P** (μ mol L^{-1})	**%N** (g g^{-1})
A. arenaria	0.05 ± 0.00	0.37 ± 0.06	0.00 ± 0.00
A. breviligulata	0.02 ± 0.00	0.29 ± 0.09	0.00 ± 0.00
**Plants**	**%C** (g g^{-1})	**%P** (g g^{-1})	**%N** (g g^{-1})
A. arenaria	50.58 ± 0.80	0.13 ± 0.01	1.33 ± 0.08
A. breviligulata	44.32 ± 2.06	0.13 ± 0.02	0.84 ± 0.12

Supplementary Table 3 | Lévy optimum calculated under a range of wind conditions and shoot numbers (N=15). The optimal clonal expansion strategy (under a range of μ values from 1.5 to 3.0 with increasing steps of 0.5) was determined for both potential area of sand deposition (**a**) and sand trapping efficiency (**b**) by comparing the mean ± SEM of the different strategies. The values in the red square (critical threshold of 55% of incoming wind) reflect average wind conditions along the Dutch coast[3]. The values to the right side indicate calmer conditions (with a lower critical threshold for sand deposition), and values to the left indicate windier conditions (with a higher critical threshold). We found total sand deposition to converge at μ ~1.5, but the number of shoots needed to reach this optimum may differ depending on the wind conditions. Similarly, sand trapping efficiency converges at μ~2 but the number of shoots required to his optimum is dependent on wind conditions. Source data are provided as a Source Data file.

a

Sand deposition

decreasing threshold for sand deposition (% incoming wind) →

increasing shoot number ↓	0.25	0.4	0.55	0.7	0.85
30	1.5-3	2.5 - 3	2 - 2.5	2	1.5
50	3	2.5	2 - 2.5	1.5 - 2	1.5
100	2.5	2	2	1.5	1.5
300	2 - 2.5	2	1.5	1.5	1.5
500	2	2	1.5	1.5	1.5
1000	2	1.5 - 2	1.5	1.5	1.5
3000	1.5 - 2	1.5	1.5	1.5	1.5
5000	1.5 - 2	1.5	1.5	1.5	1.5

b

Efficiency

decreasing threshold for sand deposition (% incoming wind) →

increasing shoot number ↓	0.25	0.4	0.55	0.7	0.85
30	3	3	3	3	3
50	3	3	3	2.5 - 3	2.5 - 3
100	3	3	3	2.5 - 3	2.5 - 3
300	2.5 - 3	2.5 - 3	2.5 - 3	2.5 - 3	2.5
500	2.5 - 3	2.5	2.5 - 3	2 - 3	2
1000	2.5 - 3	2.5	2 - 2.5	2 - 2.5	2
3000	2.5	2 - 2.5	2	2	2
5000	2 - 2.5	2 - 2.5	2	2	2

Supplementary References

1 Güsewell, S. N: P ratios in terrestrial plants: variation and functional significance. *New Phytol.* **164**, 243-266 (2004).

2 Kooijman, A., Dopheide, J., Sevink, J., Takken, I. & Verstraten, J. Nutrient limitations and their implications on the effects of atmospheric deposition in coastal dunes; lime-poor and lime-rich sites in the Netherlands. *J. Ecol.* **86**, 511-526 (1998).

3 Stepek, A. & Wynant, I. L. Interpolating wind speed normals from the sparse Dutch network to a high resolution grid using local roughness from land use maps. *KNMI, Tech. Report TR-321* (2011).
