# Supplementary material for: A Lévy expansion strategy optimizes early dune building by beach grasses
Source: Nat Commun. 2019 Jun 14;10:2656. doi: 10.1038/s41467-019-10699-8 (PMC6572860; doi:10.1038/s41467-019-10699-8)
Supplement: Supplementary file 3 — Reporting Summary [file 41467_2019_10699_MOESM3_ESM.pdf]

Corresponding author(s): v.reijers@science.ru.nl

Reporting Summary

Nature Research wishes to improve the reproducibility of the work that we publish. This form provides structure for consistency and transparency in reporting. For further information on Nature Research policies, see Authors & Referees and the Editorial Policy Checklist.

Statistical parameters

When statistical analyses are reported, confirm that the following items are present in the relevant location (e.g. figure legend, table legend, main text, or Methods section).

n/a	Confirmed	
☐	☒	The exact sample size (*n*) for each experimental group/condition, given as a discrete number and unit of measurement
☐	☒	An indication of whether measurements were taken from distinct samples or whether the same sample was measured repeatedly
☐	☒	The statistical test(s) used AND whether they are one- or two-sided<br>*Only common tests should be described solely by name; describe more complex techniques in the Methods section.*
☒	☐	A description of all covariates tested
☐	☒	A description of any assumptions or corrections, such as tests of normality and adjustment for multiple comparisons
☐	☒	A full description of the statistics including central tendency (e.g. means) or other basic estimates (e.g. regression coefficient) AND variation (e.g. standard deviation) or associated estimates of uncertainty (e.g. confidence intervals)
☐	☒	For null hypothesis testing, the test statistic (e.g. *F*, *t*, *r*) with confidence intervals, effect sizes, degrees of freedom and *P* value noted<br>*Give P values as exact values whenever suitable.*
☒	☐	For Bayesian analysis, information on the choice of priors and Markov chain Monte Carlo settings
☒	☐	For hierarchical and complex designs, identification of the appropriate level for tests and full reporting of outcomes
☒	☐	Estimates of effect sizes (e.g. Cohen's *d*, Pearson's *r*), indicating how they were calculated
☐	☒	Clearly defined error bars<br>*State explicitly what error bars represent (e.g. SD, SE, CI)*

Our web collection on statistics for biologists may be useful.

Software and code

Policy information about availability of computer code

Data collection	There was no software used for data collection.
Data analysis	R version 3.4.0 with packages lme4 and glht for experimental comparisons, and spatstat for obtaining Ripley's K;<br>ImageJ and Matlab R2515b for photo analyses;<br>Matlab R2015b for modelling, with ANSYS 17.2 for obtaining wind convolution matrix.

For manuscripts utilizing custom algorithms or software that are central to the research but not yet described in published literature, software must be made available to editors/reviewers upon request. We strongly encourage code deposition in a community repository (e.g. GitHub). See the Nature Research guidelines for submitting code & software for further information.

Data

Policy information about availability of data

All manuscripts must include a data availability statement. This statement should provide the following information, where applicable:

- Accession codes, unique identifiers, or web links for publicly available datasets
- A list of figures that have associated raw data
- A description of any restrictions on data availability

Figures 2, 4, S2, S3, S4 and S5 and Tables S1,2 are associated with raw empirical data. All obtained (survey and experimental) data and custom-made Matlab scripts will be made publically available through the DANS Easy repository upon publication.

Field-specific reporting

Please select the best fit for your research. If you are not sure, read the appropriate sections before making your selection.

☐ Life sciences ☐ Behavioural & social sciences ☒ Ecological, evolutionary & environmental sciences

For a reference copy of the document with all sections, see nature.com/authors/policies/ReportingSummary-flat.pdf

Ecological, evolutionary & environmental sciences study design

All studies must disclose on these points even when the disclosure is negative.

Study description	Field survey: We conducted a field survey on both sides of the Atlantic coast to investigate the spatial organization and expansion strategy of the two dune grass species used in this study. European marram grass (Ammophila arenaria) was sampled on the West Frysian barrier island of Schiermonnikoog, the Netherlands (53°30'25.38"N, 6°18'52.52"E) from April to June 2017. American beach grass (Ammophila breviligulata) was sampled on two barrier islands on the east coast of the United States: Hatteras island, North Carolina (35°13'58.67"N, 75°36'6.60"W) and Chincoteague island, Virginia (38° 0'25.81"N, 75°15'36.64"W) in August 2017. For both species we selected young isolated colonizing plants near the foot of the primary dunes. Earlier studies demonstrated similar tillering rates (the rate at which new shoots emerge) between species during colonization and we therefore assumed no age differences between species26. By cutting off the aboveground biomass and replacing each shoot by a labelled coloured pin, we were able to extract the spatial coordinates of all shoots (in cm) using a custom-made Matlab tool (see Supplementary Fig. 1 for a visual description of the methods). In addition, we collected soil (at ~5 cm depth in middle of clonal individual) and leaf samples (pooled per clonal individual) to assess nutrient availability.<br><br>Field experiment: We conducted a field experiment on a bare beach plain of Schiermonnikoog, the Netherlands (53°30'36.73"N, 6° 19'37.84"E) in the summer of 2016, to test the effect of the spatial shoot organization on the sand trapping ability. We constructed plots of 4 m2 in which we placed flexible artificial dune grass mimics (three plastic bristles, diameter 0.2 cm, length 75 cm, made up one shoot23) in three spatially distinct patterns (dispersed, patchy and single-patch). In total ~2000 bristles were inserted in 4 m2 PVC templates (which resulted in 500 shoots m-1, a natural shoot density previously used in biophysical studies) with the spatial patterns drilled into them and attached to wooden beams in 20 cm deep pits on the beach, after which we refilled the plots using drift-sand resulting in a canopy-height of the mimics of 55 cm. Each treatment was replicated 3 times in a randomized block design that also included a control plot (only PVC sheet, no bristles) per block, yielding 12 plots in total.<br>Sand deposition was measured every month (June, July and August) on a 0.1 x 0.1 m scale with the use of a sediment erosion bar construction.
Research sample	Field survey: We randomly selected young clonally expanding individuals growing on the foot of the dune for both ammophila species used in our study (i.e. A. breviligulata and A. arenaria).<br><br>Field experiment: Sand deposition was measured every month (June, July and August) on a 0.1 x 0.1 m scale with the use of a sediment erosion bar construction.
Sampling strategy	Field survey: The spatial shoot coordinates were extracted from still images, and subsequently used for analyses of the spatial clustering and complexity of the shoot organization. Sample size was determined following pilot experiments to test the methodology. We obtained 1471 measurements from 12 plants for A. arenaria, and 752 data points from 4 plants for A. breviligulata.<br><br>Field experiment: Sample size was determined based on pilot experiments to test the general methodology. The experiment consisted of 3 treatments (dispersed, patchy, single-patch), with 3 replicate pots (4m2 each) per treatment in a randomized block design. Sand deposition was measured every month (June, July and August) on a 0.1 x 0.1 m scale within each plot (380 points/plot) with the use of a sediment erosion bar construction.
Data collection	Field survey: VCR, LLG, AB collected the data in US. VCR and SH collected the data in NL. Shoots were labeled with pins and subsequently photographed using a leveled metal frame as a reference. Afterwards the plants were excavated and VCR noted the rhizomal connections between the labeled shoots.<br><br>Field experiment: VCR collected the data with the help of research assistants and volunteers. Using a SED erosion bar we measured the sand bed level on a 10x10 cm scale to the nearest cm.
Timing and spatial scale	European marram grass (Ammophila arenaria) was sampled on the West Frysian barrier island of Schiermonnikoog, the Netherlands

Timing and spatial scale	(53°30'25.38"N, 6°18'52.52"E) from April to June 2017. American beach grass (Ammophila breviligulata) was sampled on two barrier islands on the east coast of the United States: Hatteras island, North Carolina (35°13'58.67"N, 75°36'6.60"W) and Chincoteague island, Virginia (38° 0'25.81"N, 75°15'36.64"W) in August 2017.<br><br>Field experiment: We conducted the field experiment on a bare beach plain of Schiermonnikoog, the Netherlands (53°30'36.73"N, 6° 19'37.84"E) in the summer of 2016 with plot sizes of 4m2. Sand deposition was measured every month (June, July and August) on a 0.1 x 0.1 m scale.
Data exclusions	No data were excluded. For describing the clonal expansion strategy of individual clonal plants we selected plants with >30 step sizes to have sufficient data.
Reproducibility	Field survey: Data were collected across multiple sites within one growing season; plants were randomly selected per site.<br><br>Field experiment: The treatments were constructed in a randomized block design; measurements were repeatedly taken across the experimental period.
Randomization	Field survey: Plants were randomly selected at the foot of the primary dunes at multiple sites.<br><br>Field experiment: The treatments were set up in a randomized block design on a bare beach plane.
Blinding	Field survey: Plants were randomly selected, but during the actual measurements blinding was not applicable.<br><br>Field experiment: Treatments were set up randomly within each block. Once constructed, blinding was not applicable.

Did the study involve field work? ☒ Yes ☐ No

Field work, collection and transport

Field conditions	Fieldwork was performed during calm and dry days.
Location	Field survey: European marram grass (Ammophila arenaria) was sampled on the West Frysian barrier island of Schiermonnikoog, the Netherlands (53°30'25.38"N, 6°18'52.52"E); American beach grass (Ammophila breviligulata) was sampled on two barrier islands on the east coast of the United States: Hatteras island, North Carolina (35°13'58.67"N, 75°36'6.60"W) and Chincoteague island, Virginia (38° 0'25.81"N, 75°15'36.64"W).<br><br>Field experiment: We conducted the field experiment on a bare beach plain at the island of Schiermonnikoog, the Netherlands (53°30'36.73"N, 6°19'37.84"E).
Access and import/export	Permits for the fieldwork were obtained from Chincoteague National Wildlife Refuge (30-6-2017), Alligator River and Pea Island National wildlife refuge (14-6-2017) and Natuurmonumenten (11-4-2017).
Disturbance	No disturbance lasting beyond the actual sampling. The experiment was completely removed after the experimental period.

Reporting for specific materials, systems and methods

Materials & experimental systems

n/a	Involved in the study
☒	☐ Unique biological materials
☒	☐ Antibodies
☒	☐ Eukaryotic cell lines
☒	☐ Palaeontology
☒	☐ Animals and other organisms
☒	☐ Human research participants

Methods

n/a	Involved in the study
☒	☐ ChIP-seq
☒	☐ Flow cytometry
☒	☐ MRI-based neuroimaging
